# Effects of *Syzygium aromaticum*, *Cinnamomum zeylanicum*, and *Salvia triloba* extracts on proliferation and differentiation of dental pulp stem cells

**DOI:** 10.1590/1678-7757-2016-0522

**Published:** 2017

**Authors:** Ayşegül Mendi, Beyza Gökçınar Yağci, Mustafa Kiziloğlu, Nurdan Saraç, Derviş Yilmaz, Aysel Uğur, Duygu Uçkan

**Affiliations:** 1 Gazi University, Faculty of Dentistry, Department of Medical Microbiology, Ankara, Turkey; 2 Hacettepe University, PEDI-STEM Center for Stem Cell Research and Development, Ankara, Turkey; 3 Gazi University, Faculty of Dentistry, Department of Oral and Maxillofacial Surgery, Ankara, Turkey; 4 Mugla Sitki Kocman University, Faculty of Sciences, Department of Biology, Mugla, Turkey

**Keywords:** Dental Pulp, S. aromaticum, C. zeylanicum, Salvia triloba, Osteoblast

## Abstract

Hypersensitivity, local irritative and cytotoxic effects are known for the chemical components of *Syzygium aromaticum* and *Cinnamomum zeylanicum* contained in dental materials. However, there is no intimate data in dentistry using the whole extracts of these plants and introducing new ones. *Salvia triloba* is a well-known anti-inflammatory plant that correspondingly could be used in several dental traumas.

**Objectives:**

We aimed to show and compare the effect of *S. aromaticum*, *C. zeylanicum*, and *S. triloba* extracts on dental pulp stem cells (DPSCs) proliferation, differentiation, and immune responses.

**Material and Methods:**

Using xCELLigence, a real time monitoring system, we obtained a growth curve of DPSCs with different concentrations of the Extracts. A dose of 10 μg/mL was the most efficient concentration for vitality. Osteogenic differentiation and anti-inflammatory activities were determined by using an ELISA Kit to detect early and late markers of differentiation.

**Results:**

The level of osteonectin (ON, early osteogenic marker) decreased, which indicated that the osteogenic differentiation may be accelerated with addition of extracts. However, the level of osteocalcin (OCN, late osteogenic marker and sign of calcium granulation) differed among the extracts, in which *S. aromaticum* presented the highest value, followed by *S. triloba* and *C. zeylanicum*. Surprisingly, the determined calcium granules were reduced in *S. aromaticum* and *S. triloba*. In response to tumor necrosis factor alpha (TNF-α), *S. triloba*-treated DPSCs showed the most reduced level of IL-6 cytokine level. We suggest *C. zeylanicum* as a promising osteogenic inducer and *S. triloba* as a potent anti-inflammatory agent, which could be used safely in biocomposite or scaffold fabrications for dentistry.

**Conclusions:**

Because calcium granule formation and cell viability play a critical role in hard tissue formation, *S. aromaticum* in dentistry should be strictly controlled, and the mechanism leading to reduced calcium granule formation should be identified.

## Introduction

Medicinal plants have been extensively utilized for clinical purposes in various diseases, including dental disorders^13^. Of these, *Syzygium aromaticum*, *Cinnamomum zeylanicum*, and *Salvia triloba* are known for their antibacterial and anti-inflammatory activities. Eugenol is the main component of *S. aromaticum*, and it plays a prominent role in dental and oral preparations^13^. *C. zeylanicum* is also used in toothpastes and mouthwashes. They are used as a topical antiseptic and anti-inflammatory analgesic in dentistry. However, allergies to materials and/or extracts used in dentistry are an increasing issue and have been subject of research^11^ ^,^ ^12^ ^,^ ^16^ ^,^ ^17^.

Medications administered to teeth and oral mucosa, such as zinc oxide-eugenol (ZOE) and cinnamon, including mouthwashes, can reach the pulp tissue or periodontium after penetrating the enamel and dentin or passing through apical foramens^2^. If the medications are cytotoxic, they might disturb the function of mesenchymal stem cells (MSCs), which exist in dental pulp and in the periodontium. Therefore, it is important to study the cytotoxicity of agents used in oral treatment.

In this study, we hypothesize that a natural agent, which maintains dental pulp stem cells (DPSCs) viability, promotes osteogenic differentiation while modulating the immunological response, and that it could achieve success in regeneration during healing and may also prevent bone resorption and improve regeneration.

Although various physiological activities of the extracts have been shown, their effects on osteogenic differentiation of mesenchymal stem cells have never been assessed.

## Material and Methods

### Extraction of plant samples

*S. aromaticum*, *C. zeylanicum* bark, and *S. triloba* flower buds (named as Extracts hereafter) were purchased from a local market in Mugla, Turkey. The air-dried plant samples were extracted with ethanol (Merck, Dermstadt, Germany) using a Soxhlet apparatus. The extracts were evaporated and stored in sterile opaque glass bottles under refrigerated conditions until use. The dried extract was prepared in Dulbecco's Modified Eagle's Medium -low glucose (DMEM-LG) with 10% fetal bovine serum (FBS) (Invitrogen, Carlsbad, California, USA), 1% L-Glutamine (Sigma St. Louis, Missouri, ABD), and 1% penicillin-streptomycin (Invitrogen, Carlsbad, California, USA).

### Isolation and culture of dental pulp stem cells

Human dental pulp tissue was obtained from patients (15-20 years of age) who were undergoing extraction of third molars for orthodontic reasons at the Department of Oral and Maxillofacial Surgery, Gazi University, Ankara. All patients signed an informed consent form. After the tooth surfaces were disinfected (75% ethanol), the teeth were drilled and the dental pulp was gently extracted with forceps. The extracted pulp tissue was rinsed in a-MEM supplemented with 2 nM L-glutamine, 100 U/mL penicillin, 100 μg/mL streptomycin, and 10% FBS (Invitrogen, Carlsbad, California, USA) [hereafter referred to as the stem cell (SC) culture medium], after which it was minced into fragments of 1 to 2 mm^3^. The tissue fragments were cultured in T75 plates (Nunc, Waltham, Massachusetts, ABD) in the SC culture medium at 37°C in a humidified atmosphere containing 5% CO_2_. The cell cultures were monitored regularly with an inverted microscope (Olympos CKX41, Tokyo, Japan) and the SC culture media were changed every 3 days. After reaching 70-80% confluence, the cells were harvested with 0.25% Trypsin/EDTA (Sigma St. Louis, Missouri, USA) and sub-cultured for further experiments. The experiments were performed with passage 2-3 cells.

### Immunophenotypic analysis

The culture-expanded adherent cells were analyzed by flow cytometry (FACSARIA, Becton Dickonson, USA). The antibody panel included CD29-FITC (e-bioscience, USA), CD73-PE (BD, USA), CD90-PE (BD, USA), and CD44-PE (e-bioscience, USA) as mesenchymal stromal markers, as well as their isotype controls. CD45-FITC (BD, USA), CD14-PE (BD, USA), and CD34-FITC (BD USA) were used as hematopoietic markers to exclude cells of hematopoietic origin. The relative frequencies of the cells that expressed the respective surface markers were analyzed using FACS Diva software 6.0.0 (BD) by acquiring 10,000 events for each sample.

### Effect of *S. aromaticum*, *C. zeylanicum*, and *S. triloba* on DPSCs proliferation, using the xCELLigence system

The xCELLigence system was used according to the manufacturer's instructions. An impedance-based real time cell analyzer (RTCA), an RTCA single plate (E-plate 96), an RTCA computer, and a tissue-culture incubator constitute the xCELLigence system (Roche Applied Science, Mannheim, Germany)^15^. There are sensor electrodes on the surface of each well of the E-Plate 96. Physiologic changes in the cells are determined and measured by the electronic impedance of the sensor electrodes. Electrode geometry, ion concentration on the well, and whether the cells are attached to the electrodes affect the impedance measured between electrodes in each well. In the absence of cells, the electrode impedance is mainly determined by the ion environment at the electrode-solution interface and in the bulk solution. In the presence of cells, cells attached to the electrode sensor surfaces change the local ion environment at the electrode-solution interface, leading to increased impedance. Thus, if more cells are present in the environment growing on the electrodes, the value of the electrode impedance will be increased. This mechanism gives real time monitoring adherent cells.

To measure the background impedance, we connected the E-plate 96 to the xCELLigence system and ensured that the proper electrical contacts were established. Subsequently, 100 μl of SC culture media containing different concentrations of the Extracts (5, 10, and 25 μg/mL) and of standard culture media (control) were added to each well of E-plate 96. The cells were resuspended (5000 cells/cm^2^) in SC culture media. Cell growth and proliferation were monitored every 30 min for up to 169 h.

### Effect of *S. aromaticum*, *C. zeylanicum*, and *S. triloba* on DPSCs differentiation

The concentration that decreased the doubling time and increased the proliferation was selected based on the results from the xCELLigence system analysis. This concentration was added into the osteogenic and adipogenic differentiation media^14^. Images were obtained with a CKX41 digital imaging microscope (Olympus CKX41, Tokyo, Japan). The media were changed every 3 or 4 days for 21 days. The removed culture supernatant was stored in −80°C until the ELISA tests were conducted. Secreted osteocalcin (OCN) and osteonectin (ON) levels in culture supernatants were assessed using an ELISA kit, according to the manufacturer's instructions (R&D Systems, Inc. Minneapolis, USA). The limits of detection for the ELISA ranged from 1.2 to 75 ng/mL for OCN and 1.56 to 50 ng/mL for ON. The calcium ion concentration in the differentiation medium was measured using a QuantiChrom calcium assay kit according to the manufacturer's instructions (DICA 500, BioAssay Systems, USA).

### Determining immunomodulatory activities

DPSCs were plated at a density of 5000 cell/ cm^2^ in 96-well culture plates and allowed to attach overnight. The cells were first treated with 10 μg/mL of the Extracts for 1 h, and then 10 ng/mL of TNF-α was added. After 24 h, the cell culture supernatants were collected and stored at −80°C for use in the IL-6 and IL-10 ELISAs, according to the manufacturer's instructions. The ELISA limits were 0.052-0.118 pg/ mL for IL-6 and 0.39-25 pg/mL for IL-10. The media alone, with TNF-α, and with the Extracts were included as controls.

### Statistical analysis

All calculations were performed using the RTCA integrated software of the xCELLigence system. The RTCA software fits the curve of the selected sigmoid dose response equations to the experimental data points. The data are presented as mean (μg/mL) ± standard deviation (SD) (n=4). For the proliferation experiments, the statistical analysis was performed using one-way analysis of variance (ANOVA) (p<0.05).

## Results

### Identification of DPSCs

**Figure 1:**
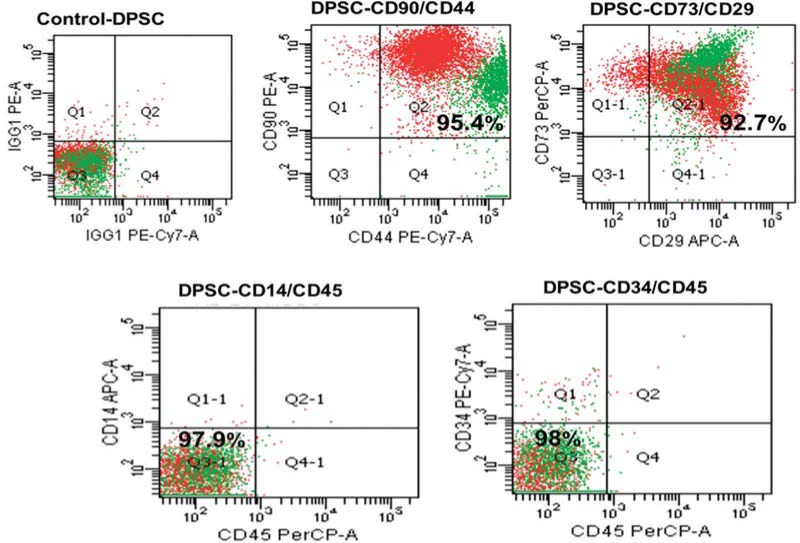
Surface markers of dental pulp stem cells (DPSCs). The cells were positive for CD90, CD44, CD29, and CD73 (mesenchymal stem cell markers) and negative for CD14, CD45, and CD34 (hematopoietic stem cell markers)

Common mesenchymal SC markers (CD29, CD73, CD44, and CD90) were consistently positive (>95%) and hematopoietic markers (CD14, CD34, and CD45) were negative (>95%) in all samples tested, indicating a mesenchymal origin of the cells (Figure 1).

### xCELLigence assays

**Figure 2:**
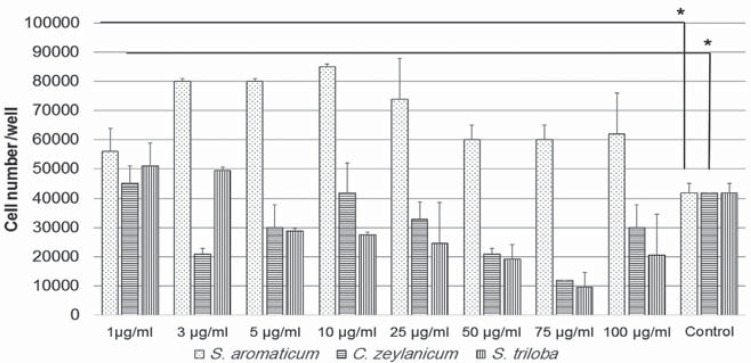
Viability of dental pulp stem cells (DPSCs) treated with different Extracts. Trypan bBue assay showed *S. aromaticum* had no toxic effect on the cells. *C. zeylanicum* and *S. triloba* reduced cell viability after 10 μg/mL. Increased concentration reduced the cell number in *S. aromaticum* and *C. zeylanicum* in a negative strong correlation

**Figure 3:**
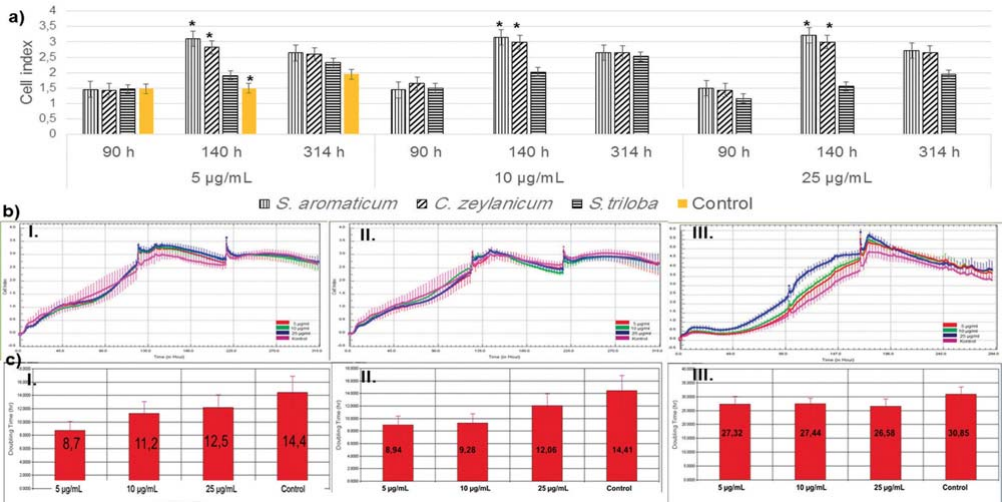
xCELLigence analysis of dental pulp stem cells (DPSCs) treated with 5, 10, and 25 μg/mL of the Extracts. a: Cell index of DPSCs at 5, 10, and 25 μg/mL; *S. aromaticum* and *C. zeylanicum* growth curve of the cells with *S. aromaticum* at 140 h (b.I); *C. zeylanicum* (b.II) and *S. triloba* (b.III) differed from the Trypan Blue assay; The doubling time of the DPSCs shortened with *S. aromaticum* (c.I), *C. zeylanicum* (c.II), and *S. triloba* (c.III)

Using Trypan Blue, we found that only the *S. aromaticum* extract induced cell proliferation (Figure 2). 5, 10, and 25 μg/mL concentrations were selected for xCELLigence analysis according to the highest cell number at the end of the culture period. The increase in cell proliferation with *S. aromaticum* and *C. zeylanicum* was in a positive correlation with the Control Group at 140 h (Figure 3a). Growth curves were obtained in the real time monitoring system (Figure 3b.I-III). The doubling time (DT) of the Extracts suggested that *S. aromaticum* (Figure 3c-I) and *C. zeylanicum* (Figure 3c-II) reduced the DT in a dose-dependent manner. *S. triloba* (Figure 3c-III) showed a reduction in DT independent from the concentrations. We calculated the IC50 values, which were 8001 μg/mL, 102 μg/mL, and 1475 μg/mL for *S. aromaticum*, *C. zeylanicum*, and *S triloba*, respectively. The 10 μg/mL concentration was selected for further studies.

### Differentiation assays of DPSCs

**Figure 4:**
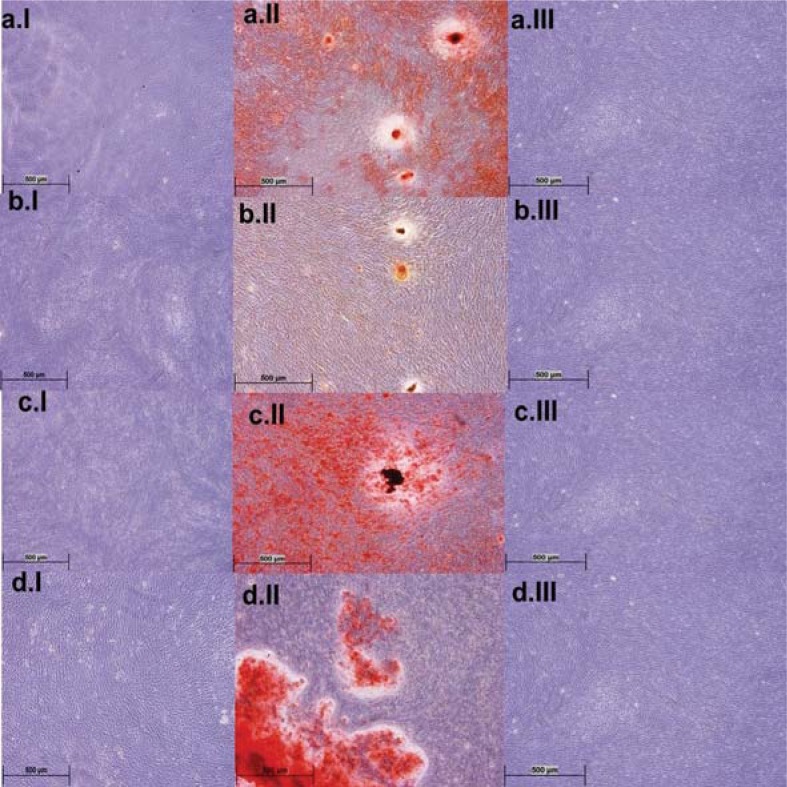
Potential differentiation of dental pulp stem cells (DPSCs) referred as Control Group (a.I-III); adipogenic differentiation was not shown in DPSCs Control Group (a.I) and in the Extract Group (b-d.I). Osteogenic differentiation was observed in Control Group (a.II) with calcium granules and extracellular matrix. Calcium granules are seen as black nodules and the extracellular matrix was dyed red. The extract of *S. aromaticum* (b.II) reduced the dying of extracellular matrix and formation of calcium granules. *C. zeylanicum* (c.II) induced calcium granules and extracellular matrix formation, while *S. triloba* (d.II) showed reduced effect. The cells with SC medium are shown in a-d.III (4x, Olympus CKX41, Tokyo, Japan)

**Figure 5:**
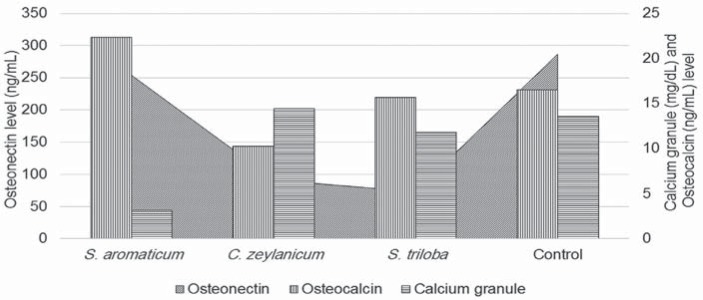
Calcium granule, osteocalcin, and osteonectin levels of dental pulp stem cells (DPSCs) treated with the Extracts

Adipogenic differentiation was not seen in the Control (Figure 4a.I) and in the Extract Group (Figure 4b-d.I). Morphologically, approximately 20% of the cells adopted rounder shape in all Groups. However, no lipid droplets were observed. On the other hand, the DPSCs in the Control Group were well differentiated in osteogenic differentiation media (Figure 4a.II). Calcium granules were seen as dark nodules, and the extracellular matrix was dyed red. *S. aromaticum*-treated cells showed reduced calcium granules and extracellular matrix staining (Figure 4b.II). *C. zeylanicum-*treated cells presented remarkable calcium granules and extracellular matrix dying (Figure 4c.II). *S. triloba* displayed an average osteogenic differentiation, and some clones reacted with the Alizarin Red S. The quantity of calcium granules and extracellular matrix protein levels is shown in Figure 5. Osteonectin levels showed a decrease in cells treated with the Extracts. High calcium concentration as a measure of osteogenic differentiation was supported by microscopic analysis. *S. aromaticum* showed the lowest calcium concentration, while *C. zeylanicum* presented the highest. Interestingly, regarding osteocalcin levels, *S. aromaticum* and *S. triloba* presented high sign of granulation, but low calcium concentration. However, we found no statistical significance on this difference.

### Determining the preventive effect of plant extracts on the inflammatory response of DPSCs following TNF-α stimulation

**Figure 6:**
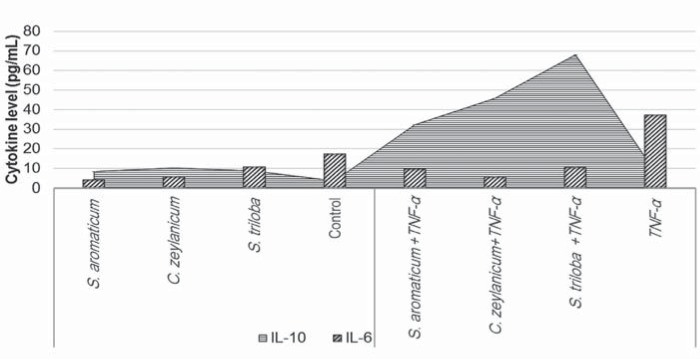
IL-6 and IL-10 levels determined in the culture supernatant of dental pulp stem cells (DPSCs). IL-10 level reached 67.9 pg/mL with *S. triloba* in response to TNF-α induction

Both IL-6 and IL-10 were present in DPSCs cell culture supernatants (Figure 6). When the Extracts were used alone, IL-6 levels were found decreased compared to the Control Group. We observed increased IL-6 levels after TNF-α induction. The Extracts were able to prevent the increase of IL-6 levels in response to TNF- α induction. An anti-inflammatory effect was observed in the presence of *S. triloba*, *C. zeylanicum*, and *S. aromaticum*, respectively, but there was no statistical significance.

## Discussion

In this study, we presented the effects of *S. aromaticum*, *C. zeylanicum*, and *S. triloba* extracts on DPSCs. Using real time monitoring and quantitative analyses, we showed that *C. zeylanicum* and *S. triloba* extracts have positive effects on human DPSCs.

Initially, we aimed to define the effective concentration of the plant extracts. Oriental medicine practices are primarily based on personal experience, but often rely on unknown mechanisms, resulting in difficulties in dose specification. The xCELLigence system is a sophisticated cell-based assay. It continuously monitor cell proliferation, viability, and label-free cytotoxicity in real time, by showing the physiologic state of the cells and eliminating expensive reagents, which are used in conventional cell analyses^9^. We found a reduced cell index in a ratio of 71% for *C. zeylanicum* and *S. triloba* in the Trypan Blue assay at 5 μg/mL, while an increase with a proportion of 47% and 21% was seen in the xCELLigence assay. Growth curves provide information on three parameters: the lag phase before cell proliferation is initiated after subculture, the DT in the middle of the exponential growth phase, and the terminal density. A shortened DT at 5 and 10 μg/mL was observed with *C. zeylanicum* and *S. aromaticum*, suggesting cell proliferation would increase more rapidly and the healing of the fracture or implant region could be shortened. It is also important to determine IC50 values, since the IC50 represents the concentration of a drug that is required for 50% inhibition *in vitro*. The IC50 for *S. aromaticum* (8001 μg/mL), *C. zeylanicum* (102 μg/mL), and *S. triloba* (102 μg/mL) was calculated and compared with the literature. Surprisingly, the clinically used eugenol/*S. aromaticum* concentration was 6.5 M^1^, which is >650000 times the effective concentration (10 μg/mL) and ≥812 times the IC50 value calculated in this study. The wide difference between the data lead us to suggest that *S. aromaticum*/eugenol could penetrate pulp tissue and trigger adverse effects, such as cell cytotoxicity. Eugenol containing endodontic cements (zinc oxide-eugenol) is applied to a variety of dental tissues. When placed onto a wet soft tissue, a much greater amount of eugenol would release from ZOE onto dentin, and the released eugenol is considered one of the ingredients responsible for cytotoxicity and allergic reactions^10^. Thus, the release of eugenol/*S. aromaticum* should be controlled, and an alternative natural agent should be tested to find whether it has similar properties to *S. aromaticum*.

To show whether the extracts induce differentiation of DPSCs, we constructed an *in vitro* model in which lipid-laden adipocytes, calcium granules, and early and late markers of osteogenesis were examined after 21 days. At the end of the culture period, adipogenic differentiation was not seen in DPSCs. Our findings are in line with those of Gronthos, et al.^7^ (2000), who expanded DPSCs from single-cell clones and demonstrated that these cells presented osteogenic differentiation, but do not form lipid-laden adipocytes. The osteogenic differentiation potential of DPSCs *in vitro* and *in vivo* has been well-documented in several studies^3^ ^,^ ^7^. During osteogenic cell differentiation, the markers of undifferentiated cells are gradually turned off, and differentiation markers are sequentially expressed. We observed sequential secretion of proteins at the end of the assay, in which the ON levels decreased in the Extract-treated group, compared to the control group. ON is an early marker of osteogenesis that is synthesized by preosteoblasts and has less affinity to collagen. The ON transcript is relatively stable, with a half-life of >24 hours under conditions of transcription arrest^4^. DPSCs treated with the Extracts showed reduced ON and increased OCN levels, while the Control group was *vice versa*, suggesting that osteogenic differentiation was more rapid in the Extract-treated cells. However, conspicuously, calcium concentrations were low in *S. aromaticum* and *S. triloba*-treated DPSCs, despite the increased OCN levels. *S. aromaticum* and *S. triloba* may disturb the calcium deposit development of the cells. Similarly, Anpo, et al.^1^ (2011) provided evidence that eugenol/*S. aromaticum* reduces collagen synthesis, which play a critical role in osteogenesis. Because *S. aromaticum*/eugenol has been extensively used in dental practice as an endodontic medication and for prevention of dry socket, further molecular studies should be done to clarify its effect on osteogenic differentiation.

Oral surgery procedures are frequently associated with locoregional inflammatory processes. Nonsteroidal anti-inflammatory drugs (NSAIDs) and glucocorticoids are commonly prescribed as anti-inflammatory agents after oral and maxillofacial surgery. These compounds have no deleterious effect on bone cells. However, long-term use of NSAIDs has repeatedly been reported to interfere with bone remodeling and to delay bone healing^6^. Indications, choice of compound dosage, duration of treatment, precautions for use, drug interactions, contraindications, adverse events, and the risk of infection related to the use of an anti-inflammatory drug are still controversial^8^. Therefore, in diabetic or immunocompromised patients, agents with anti-inflammatory activity, which improve bone healing without masking infection, are desired^5^. In our study, both IL-6 and IL-10 were present in the DPSCs cell culture supernatants. When the Extracts were used alone, the IL-6 level was decreased in DPSCs. A reduced IL-6 level in response to TNF-α showed that the plant extracts could serve as immunomodulatory agents in inflammatory conditions. *S. triloba* showed a positive anti-inflammatory effect in response to TNF-α stimulation. We therefore believe that it could be a natural alternative to NSAIDs and glucocorticoids.

In this study, we determined that *C. zeylanicum*, as a promising osteogenic inducer, and *S. triloba*, as a promising anti-inflammatory agent, could be used individually or in combination with biocomposites or scaffold fabrications in dentistry. We showed the evidence that *S. aromaticum*/eugenol reduced the osteogenic differentiation of DPSCs. Because eugenol has been extensively used in dental practice as an endodontic medication, we suggest that the concentration of eugenol should be controlled and that alternative agents should be used. Further studies to determine the mechanism of the adverse effects of eugenol are necessary to reduce the toxic and antiosteogenic effect and to prevent the failure of endodontic treatments. This study showed the biological effect of whole plant extracts. Obviously, the chemical composition and the fractions of the extract should be determined.
